# Herat’s Catastrophe: Earthquakes Deepen Afghanistan’s Healthcare Crisis

**DOI:** 10.31662/jmaj.2024-0002

**Published:** 2024-09-27

**Authors:** Mirwais Ramozi, Hosain Barati, Yudai Kaneda, Akihiko Ozaki, Yasuhiro Kotera

**Affiliations:** 1 Faculty of Medicine, Kateb University, Kabul, Afghanistan; 2 School of Medicine, Hokkaido University, Sapporo, Japan; 3 Department of Breast and Thyroid Surgery, Jyoban Hospital of Tokiwa Foundation, Iwaki, Japan; 4 Faculty of Medicine and Health Sciences, University of Nottingham, Nottingham, United Kingdom

**Keywords:** Earthquakes, Afghanistan, Disaster, Public Health, Politics

## Abstract

In October 2023, Herat Province in Afghanistan was devastated by three earthquakes, resulting in 1,480 fatalities and 1,950 injuries, affecting approximately 154,000 people. The destruction included over 21,300 buildings, including 40 healthcare facilities, intensifying an existing humanitarian crisis under Taliban rule since August 2021. A comprehensive and coordinated response is vital for sustainable recovery and resilience, transcending political barriers to address the immediate and long-term needs of the Afghan population.

In October 2023, Herat Province in Afghanistan was struck by three earthquakes of magnitude 6.3, resulting in significant casualties and widespread destruction. The first two quakes had the most severe impact, with 1,950 injuries and 1,480 fatalities reported. These events directly affected around 43,400 individuals in six regions, destroying 3,330 homes and causing damage to approximately 21,300 buildings, including vital infrastructure ^(1)^. The United Nations Office for the Coordination of Humanitarian Affairs estimates that 154,000 people have been affected, and the WHO highlights that over 114,000 require emergency medical aid, including 7,500 pregnant women. The WHO’s assessments reveal that over 40 healthcare facilities have been damaged, with a risk of collapse looming over others ^(2)^.

These earthquakes have further strained the already critical humanitarian crisis in Afghanistan, which is exacerbated by Taliban’s rule established in August 2021. The Taliban’s ban on female NGOs has led to the withdrawal of crucial aid groups, halting essential aid programs and exacerbating the shortage of medicines, medical equipment, and essential healthcare services. The subsequent closure of many health centers has significantly limited access to healthcare, disproportionately affecting women and children. These factors have contributed to high malnutrition rates, food insecurity, and hindered livelihoods. The healthcare sector’s collapse has been especially detrimental to maternal and neonatal health, with high rates of complicated deliveries and mortality ^(3), (4)^.

The aftermath of the earthquake presents additional challenges: a scarcity of healthcare facilities, clean water, food, and adequate shelter, and an increased risk of contagious diseases in overcrowded camps, all amidst the onset of colder weather ^(2)^. These conditions underline the need for comprehensive initiatives to mitigate disaster consequences and enhance future preparedness. Despite ongoing aid efforts, there is an urgent demand for a coordinated international humanitarian response to assist those affected in Herat. Priorities must include delivering emergency medical aid, ensuring access to shelter, clean water, and sanitation, and addressing food security and nutritional needs. Infrastructure rehabilitation and disaster preparedness are critical to long-term resilience. This necessitates substantial improvements to the healthcare system and reinforces international cooperation regardless of the political issues ^(5)^.

In conclusion, the dual crises of political turmoil and natural disasters in Herat Province necessitate a robust and multifaceted international response. Omelicheva’s research reports the impacts of various types of natural disasters, such as droughts, earthquakes, and floods, and their interaction with political instability on nations; for instance, the 2004 tsunami had differing impacts on Indonesia and Sri Lanka, highlighting how natural disasters can either promote peace or exacerbate conflict depending on the political backdrop ^(6)^. However, in the present case, the lack of preparedness and coordination among healthcare systems has been evident in Afghanistan, exacerbating the crisis triggered by natural disasters and political instability. In Haiti as well, despite significant international aid following the 2010 earthquake, the country continues to struggle with effective governance, further hindered by political crises ^(7)^. Therefore, immediate relief must be paired with strategic investments in infrastructure and healthcare to foster sustainable recovery and resilience. The international community must act swiftly and compassionately to address the needs of Afghan people, ensuring a comprehensive response that transcends temporary relief.

## Article Information

### Conflicts of Interest

Dr Ozaki reported personal fees from Medical Network Systems Inc. and Kyowa Kirin co. ltd. outside the submitted work. No other conflicts of interest were reported.

### Author Contributions

Conception and Design of the Study:

Mirwais Ramozi and Hosain Barati

Writing the Paper:

Mirwais Ramozi and Hosain Barati

Clitical Revision of the Paper:

Yudai Kaneda, Akihiko Ozaki and Yasuhiro Kotera

All the authors read the final draft and approved submission.
